# 46,XX Male Disorder of Sexual Development: A Case Report

**DOI:** 10.4274/Jcrpe.1098

**Published:** 2013-12-10

**Authors:** Ahmet Anık, Gönül Çatlı, Ayhan Abacı, Ece Böber

**Affiliations:** 1 Dokuz Eylül University Faculty of Medicine, Department of Pediatric Endocrinology, İzmir, Turkey

**Keywords:** Disorder of sexual development, XX male, SRY gene

## Abstract

The main factor influencing sex determination of an embryo is the sex-determining region Y (SRY), a master regulatory gene located on the Y chromosome. The presence of SRY causes the bipotential gonad to differentiate into a testis. However, some individuals carry a Y chromosome but are phenotypically female (46,XY females) or have a female karyotype but are phenotypically male (46,XX males). 46, XX male is rare (1:20 000 in newborn males), and SRY positivity is responsible for this condition in approximately 90% of these subjects. External genitalia of 46,XX SRY-positive males appear as normal male external genitalia, and such cases are diagnosed when they present with small testes and/or infertility after puberty. Herein, we report an adolescent who presented with low testicular volume and who was diagnosed as a 46,XX male. SRY positivity was demonstrated in the patient by fluorescence in situ hybridization method.

**Conflict of interest:**None declared.

## INTRODUCTION

46,XX male, namely testicular disorder of sexual development (DSD), is a rare clinical condition with a reported incidence of 1:20 000 in newborn males. Although clinical signs may be heterogeneous, external genitalia appear to be completely virilized in 90% of 46,XX males. These cases are usually diagnosed after puberty when they present with hypogonadism, gynecomastia, and/or infertility (1). In the majority of these cases, Y chromosome is translocated on X chromosome as the result of recombination in the distal parts of short arms of X and Y chromosomes during paternal meiosis (SRY-positive). Hypospadias, undescended testes, or various degrees of inadequate virilization in the external genitalia are seen in 10% of the cases that do not carry Y chromosome [sex-determining region Y (SRY)-negative]. Short stature and normal mental development are the other clinical characteristics of 46,XX males (2).

Differentiation from urogenital fold into bipotential gonad is the initial stage of gonadal differentiation and sexual development in the human. The SRY gene has the key role in differentiation of the bipotential gonad into testis. The etiology of 46,XX male DSD is not well defined. Nevertheless, Y translocation including SRY gene has been reported in the majority of these patients. Mutations in undefined genes and Y mosaiism are blamed to be the other likely mechanisms in SRY-negative patients (1). Although the SRY gene appears as the major regulator in testicular differentiation, many genes functioning in this pathway have been defined (1).

This report describes an adolescent who was diagnosed as a case of SRY-positive 46,XX male DSD. The patient presented with small testes but no signs of undervirilization. 

## CASE REPORT

A 16-year-old male presented with small testis size. The medical history revealed that he had been followed for retractile testis until the age of 8 years. He was referred by his physician for the absence of increase in testicular size. The patient was born at term after an uneventful pregnancy, and there was no parental consanguinity. His body weight was 57 kg [(-0.49 standard deviation score (SDS)], height was 175 cm (+0.14 SDS). Axillary hair was normal for an adult male, pubic hair was Tanner stage IV, stretched penile length was 11 cm, and testicular volume in both testicles was 3 mL. The results of laboratory analyses were as follows: basal follicle-stimulating hormone: 42 mIU/mL (N: 1.7-10.3), luteinizing hormone: 7.6 mIU/mL (N: 0.4-7.0), and testosterone: 2.6 ng/mL (N: 2.0-6.2 mg/mL). Free thyroxine was 1.02 ng/dL (N: 0.7-1.8) and thyroid-stimulating hormone was 1.7 µIU/mL (N: 0.35-4.94). Due to hypergonadotropic hypogonadism, karyotype analysis was performed in two different laboratories, and 46,XX was detected. Fluorescence in situ hybridization (FISH) analysis showed that SRY locus had been translocated to the short (p) arm of the X chromosome. Computed tomography of the pelvis revealed a normal prostate gland and normal seminal vesicles. On semen analysis, the semen sample was translucent and contained no sperms (azoospermia). A testosterone replacement therapy, in a dose of 200 mg per month, was initiated.

## DISCUSSION

The first 46,XX male DSD patient was reported in 1964 by de la Chapelle et al (3). Although 46,XX male DSD is frequently sporadic (4), familial cases have also been reported (5). The present case was considered sporadic since he had no family history.

At least three mechanisms have been suggested for the etiology of 46,XX male DSD: (i) Translocation of Y chromosome including the SRY gene on the X chromosome or on autosomal chromosomes, (ii) X-linked mutation/overexpression in the genes that cause testis differentiation or mutation/overexpression in autosomal genes [e.g. SRY box-related gene 9 (SOX9)] in SRY negative XX males, and (iii) secret Y mosaicism found only in the gonads (1).

46,XX male patients can be classified into two groups according to presence (90% of the cases) or absence (10% of the cases) of SRY gene (1,6). Translocation of Y chromosome including the SRY locus on X chromosome, which occurs due to recombination during paternal meiosis, can be easily demonstrated via molecular analyses (FISH and PCR) in 90% of 46,XX male DSD cases (4,7,8,9). In the present case, demonstration of SRY locus translocated on the short arm of X chromosome via FISH method supports the view that SRY is an important factor for male differentiation. Appearance of the external genitalia and masculinization are usually normal in 46,XX SRY-positive males (2). 

Before puberty there is no clinical sign except for undescended testis and therefore 46,XX SRY-positive males are usually diagnosed in late adolescence or adulthood through chromosome analyses performed for infertility and/or small testis (10). In the present case, the external genitalia was completely male, pubic hair and penile size were normal. Small testis size was the presenting complaint.

46,XX SRY-negative males can be diagnosed just after birth because of inadequate virilization of external genital organs (2). Although incomplete virilization of external genitalia is common in 46,XX SRY-negative males, complete masculinization and normal male appearance of external genitalia can also be seen (5). SOX9 normally functions in testis differentiation together with SRY, and duplication of this gene may lead to development of SRY-negative 46,XX male. Recently, duplication and triplication of SOX9 and duplication of SOX3 gene that leads to 46,XX SRY-negative males have been reported (11,12,13). In addition, mutations in encoding R-spondin1 (RSPO1) lead to an extremely rare clinical syndrome that combines SRY-negative XX male sex reversal with palmoplantar hyperkeratosis, and a predisposition to squamous cell carcinoma of the skin (14). Also, it has been reported that Y mosaicism in SRY-negative cases may also be another cause of XX male development (15). Since the present case was found to have SRY positivity, further analysis for SOX9, SOX3 and RSPO1 genes or for Y mosaicism were not performed.

While testosterone levels of 46,XX male DSD cases are normal at puberty, testosterone synthesis is impaired in adulthood. Gonadotropin levels exceed normal limits in mid-puberty resulting in hypergonadotropic hypogonadism, which is a typical laboratory finding in adulthood (2,16). Testicular volumes are usually less than 5 mL in these cases. While testis morphology is normal in infancy, hyalinization of the seminiferous tubules in early childhood causes loss of spermatogonia (7,16). Ergun-Longmire et al (2) reported age-appropriate gonadotropin and testosterone levels in 4 prepubertal XX male cases in the mini-puberty period (2). Normal virilization and testosterone level of the present case in puberty are coherent with previous reports and suggest that such cases have adequate gonad function so as to start puberty. Testosterone replacement should be given to the cases which have clinical and/or laboratory signs of androgen deficiency in puberty. Providing physiological orientation is important to preclude social and sexual problems, surgical repair is recommended as soon as possible in cases with external genital anomaly (1). In the present case, testosterone replacement was started for treatment of hypergonadotropic hypogonadism.

In conclusion, 46,XX male DSD should also be considered in the differential diagnosis of cases whose testicular volumes do not increase in puberty and/or who present with ambiguous genitalia in early childhood. 
